# Development of the Labor Pain Relief Attitude Questionnaire for pregnant women (LPRAQ-p)

**DOI:** 10.1186/s12884-020-03415-8

**Published:** 2020-11-23

**Authors:** Lianne P Hulsbosch, Ivan Nyklíček, Eva S Potharst, Myrthe GBM Boekhorst, Victor JM Pop

**Affiliations:** 1 Center of Research in Psychological and Somatic disorders (CoRPS), Department of Medical and Clinical Psychology, Tilburg University, P.O. BOX 90153, 5000 LE Tilburg, the Netherlands; 2 UvA minds, University of Amsterdam, Amsterdam, the Netherlands; 3 Research Institute of Child Development and Education, University of Amsterdam, Amsterdam, the Netherlands

**Keywords:** Attitude, Labor pain relief, Epidural analgesia, Antenatal, Validation

## Abstract

**Background:**

Receiving epidural analgesia during labor can possibly have negative consequences for mother and child. Yet, the use of epidural analgesia rapidly increased in the Netherlands over the last decade. Since antenatal plans for labor pain relief have been related to epidural analgesia use during labor, the aim of the current study was to develop a Labor Pain Relief Attitude Questionnaire for pregnant women (LPRAQ-p).

**Methods:**

Three focus group interviews were conducted with pregnant women, new mothers and caregivers and 13 candidate items were derived. Psychometric properties were tested with explorative factor analysis in sample I (*N* = 429) and a subsequent confirmatory factor analysis in a different sample II (*N* = 432).

**Results:**

The explorative factor analysis suggested a two-factor seven-item solution: a ‘women’s perception’ and ‘social environment’ subscale. The confirmatory factor analysis confirmed an excellent six-item model fit with appropriate internal consistency. Higher scores on the six-item LPRAQ-p indicate greater willingness for request of pain relief medication during labor. Two-tailed *t*-tests showed that women with elevated levels of depression and pregnancy-specific distress symptoms, nulliparous women and multiparous women with complications during a previous delivery had greater willingness for request of pain relief medication during labor. Linear regression showed that the most important association with higher scores on the LPRAQ-p were high pregnancy-specific distress symptoms.

**Conclusions:**

This study showed the LPRAQ-p to be a valid instrument to evaluate attitude towards labor pain relief in pregnant women. High scores on this questionnaire are associated with high levels of pregnancy-specific distress symptoms.

## Background

Labor pain is an inevitable part of childbirth, and is one of the most severe types of pain a woman will endure in comparison to other painful experiences [1, 2]. Labor pain consists of both visceral and somatic pain [3], and its severity is associated with the intensity, duration and frequency of the uterine contractions and increases with greater cervical dilatation [4, 5]. Labor pain is a complex phenomenon involving sensory, emotional and cognitive factors [5]. Especially the cognitive factor, i.e. the meaning attached to pain during labor and the expectations with regard to this pain, is crucial to how women experience labor, since it determines their coping behavior and the extent to which they can successfully adapt to labor pain [6].

Antenatal expectations of labor pain are important in how pregnant women wish to manage their pain in labor [7]. However, it is difficult for pregnant women to imagine what labor pain would feel like, even for multiparous women it is difficult to recall the labor pain they already have experienced before [8]. To avoid excessive pain in labor and to be able to cope with the pain, many women request epidural analgesia (EA) for pain relief during labor, which is often planned during pregnancy [9, 10]. Several factors have been associated with the request for pain relief during labor, such as antenatal pain catastrophizing [11], antenatal fear of childbirth [12–14], external locus of control [15], prior epidural [16] and nulliparity [10, 17]. It is reasonable to believe that previous childbirth experiences also play a role, as well as antenatal depression. Depression has been associated with expectations for negative outcomes in general [18, 19], which could imply that pregnant women with depressive symptoms may have worse labor pain expectations and therefore could have greater willingness for request of pain relief medication during labor. Besides these personal factors influencing a woman’s decision to request for pain relief during labor, a woman’s attitude towards labor pain relief is influenced by cultural background, antenatal caregivers and social environment such as partner, family and friends [4, 9].

Although pain intensity scores are lower in women who receive EA during labor, a recently updated Cochrane review provided insight in the adverse effects of EA [20]. Women who receive EA are more likely to have a prolonged first and second stage of labor and an increased need for additional oxytocin [20]. Hypotension, motor blockade, fever and urinary retention have been related to EA as well [20]. EA was found to be associated with a heightened risk of instrumental delivery, but when only considering trials performed after 2005 this association was annihilated [20]. However, a recent study found an association between the duration of exposure to EA during labor and non-spontaneous births [21].

In the Netherlands, the use of EA rapidly increased in the last decade to 21.5% [22], but is still low compared to other Western countries like Finland, Belgium and the USA (adjusted for parity, between 68,9% and 71.0%) [23]. Considering this increase in EA rate and the possibly negative consequences EA may have, it is important to gain more insight into a woman’s attitude towards labor pain relief. It is especially important to obtain this knowledge in the antenatal period, since antenatal plans for EA are highly associated with receiving EA during labor [9]. Also, pregnant women who plan to have EA were found to receive EA earlier in labor than women who prefer to avoid pain relief [9]. As far as we know, no questionnaire has been developed that measures a pregnant woman’s attitude towards labor pain relief, following a strict methodological protocol including focus group interviews followed by explorative factor, reliability and confirmatory factor analyses [24]. Therefore, the primary aim of the current study was to construct a Labor Pain Relief Attitude Questionnaire for pregnant women (LPRAQ-p). The secondary aim was to investigate the reliability, concurrent and construct validity of this new instrument. With regard to construct validity, we aimed to examine possible differences in scores on this questionnaire regarding depression symptoms, pregnancy-specific distress symptoms, parity and history of complications during a previous delivery.

## Methods

### Procedure

To gather information on the issues that were of importance to labor pain relief attitude, we formed three focus groups for in-depth interviews: a group of six pregnant women (three nulliparous and three multiparous women), a group of six women who had recently given birth, and a group of 12 obstetric caregivers (six midwives and six maternity nurses). During the focus group interviews, the participants discussed topics that were important to a pregnant woman’s attitude towards labor pain relief. The interviews took place in the office of one of the participating midwives and were supervised by research staff from the university (two psychologists, a midwife and a senior staff member (VP)). Transcriptions were made of the recorded interviews. An expert panel evaluated the transcribed interviews, discussed possible candidate items and omitted double items until a consensus was reached, resulting in 13 candidate items. A five-point Likert scale was used to format the items. The scale ranged from 1 (‘completely disagree’) to 5 (‘fully agree’), with higher scores reflecting greater willingness for request of pain relief medication during labor. This first version of the LPRAQ-p was then distributed and completed by women at 32 weeks of pregnancy, as part of a large population-based cohort study, the Holistic Approach to Pregnancy and the first Postpartum Year (HAPPY) study, the design of which is published elsewhere [25]. The HAPPY study was approved by the Psychology Ethics Committee at Tilburg University (protocol number EC-2012.25) and reviewed by the Medical Ethical Committee of the Máxima Medical Centre Veldhoven. Written informed consent was obtained from all women included in the study.

### Participants

Between December 2012 and December 2013, a total of 861 women completed the 13-item LPRAQ-p at 32 weeks of pregnancy, and this group of women was randomly separated into a sample I and II by SPSS. While sample I (*N* = 429) was used to perform an exploratory factor analysis (EFA) and reliability analysis, data of sample II (*N* = 432) was used to conduct a confirmatory factor analysis (CFA). Sample I and II met the criteria to conduct factor analyses, namely four to ten participants per item and a minimum of 100 participants [24]. Because the characteristics of the two samples were identical, the data of both samples were subsequently combined for assessment of the concurrent and construct validity.

#### Measurements

Besides the 13-item version of the LPRAQ-p, the women completed the Dutch version of the Edinburgh Depression Scale (EDS) [26] and the Tilburg Pregnancy Distress Scale (TPDS) [27] at 32 weeks of pregnancy. In addition, lifestyle features such as *smoking* (yes/no) and *alcohol intake* (yes/no) during pregnancy were obtained at 32 weeks of pregnancy. Several demographic, psychological and obstetric parameters were collected at baseline at 12 weeks of pregnancy. These parameters included *age*, *level of education* (low or medium/high (high = Bachelor’s or Master’s degree)), having a *paid job* (yes/no), *living with a partner* (yes/no), *depressive episode earlier in life* (yes/no), *parity* (primiparous/multiparous), *unplanned pregnancy* (yes/no) and *problems with previous delivery* (yes/no). Problems with previous delivery were for instance delayed dilation phase, secondary Caesarean section, use of ventouse or forceps, fetal hypoxia, prolonged second stage of labor, primary Caesarean section and fetus in breech position.

#### EDS

The Dutch version of the EDS measured symptoms of depression during pregnancy. This 10-item questionnaire is validated in both postpartum [28, 29] and pregnant women [26]. A cut-off score of 10 has been described in the third trimester [26]. Total scores range from 0 to 30, with higher scores reflecting more depression symptoms. The EDS is a reliable instrument to screen for symptoms of depression in each trimester of pregnancy, with Cronbach’s alpha’s being 0.82, 0.83 and 0.84 per trimester respectively [26]. In the current study, the Cronbach’s alpha in the third trimester of pregnancy was 0.83.

#### TPDS

 Worry symptoms about pregnancy and delivery were measured using the 11-item negative affect (TPDS-NA) subscale of the TPDS [27]. The TPDS-NA subscale consists of three subcomponents, of which one was used in the current study, namely the five-item subcomponent regarding worries about delivery [27, 30]. The total score of the TPDS-NA subscale ranges from 0 to 33, with higher scores reflecting higher levels of pregnancy-specific distress. The TPDS has been shown to be a valid and reliable instrument in Dutch pregnant women, with a Cronbach’s alpha of 0.81 for the TPDS-NA [27, 30]. The Cronbach’s alpha for the TPDS-NA in the current study was 0.76. In a review, the internal consistency and structural validity of the TPDS were evaluated as excellent [31].

### Statistical methods

The Statistical Package for Social Sciences (SPSS version 24, IBM, Chicago IL, USA) was used to perform statistical analyses. AMOS (version 24, IBM, Chicago, IL, USA) was used to carry out CFA.

#### Factor analyses

 EFA was conducted on the first 13-item version of the LPRAQ-p in Sample I. For factor retention, we used a principal component analysis with scree plot, where we considered factor loadings above 0.40 to be significant. Items that loaded on more than one dimension were retained when the difference exceeded 0.20. Internal consistency was assessed by Cronbach’s alpha for the total LPRAQ-p and its possible subscales obtained from factor analysis.

Subsequently, the stability of the factor structures, that were determined with EFA, was tested by performing CFA on the refined version of the LPRAQ-p in sample II. The comparative fit index (CFI), normed fit index (NFI), Tucker-Lewis Index (TLI), and the root mean square error of approximation (RMSEA) with lower bound were determined to evaluate model fit. We considered the model fit to be excellent with a CFI ≥ 0.80, NFI ≥ 0.80, TLI ≥ 0.80, and RMSEA ≤ 0.05 with an appropriate lower bound set at 0.04 [32, 33].

#### Concurrent and construct validity

 We examined the concurrent validity by correlating the LPRAQ-p with previously validated measures: the EDS, the TPDS-NA and the worries about delivery subcomponent of the TPDS-NA (Pearson’s *r* correlations, two-tailed). We used hypothesis testing to evaluate the construct validity of the LPRAQ-p by using two-tailed *t*-tests to compare its scores in various subgroups of women (women with and without elevated levels of depression and pregnancy-specific distress symptoms, nulliparous and multiparous women, and multiparous women with or without reported complications during a previous delivery). Effect sizes were calculated with regard to Cohen’s *d* (0.20 = small, 0.50 = medium, and 0.80 = large) and *r* (0.10 = small, 0.30 = medium, and 0.50 = large) [34]. The following hypotheses were tested: (i) women with elevated levels of depression and pregnancy-specific distress symptoms will have higher scores on the LPRAQ-p than women without elevated levels of depression and pregnancy-specific distress symptoms, (ii) nulliparous women will score higher on the LPRAQ-p compared to multiparous women, (iii) multiparous women without serious complications during a previous delivery will have lower scores on the LPRAQ-p compared to those with complications.

Finally, a multiple linear regression analysis was performed with scores on the LPRAQ-p as dependent variable and depression and pregnancy-specific distress symptoms as independent variables, after adjustment for the following three covariates: age, level of education and parity. We entered depression symptoms and the three covariates in step 1 and entered pregnancy-specific distress symptoms in step 2. This resulted in a final model with five predictors.

## Results

### Factor analyses

The women in samples I and II had similar characteristics (Table 1). All item scores were normally distributed in sample I. Items 2 and 5 were eliminated based on face validity. The Kaiser-Meyer-Olkin index was greater than 0.60 (0.80) and the Bartlett’s test of sphericity value was significant (*p* < 0.001). The scree plot suggested a two-factor solution with a total explained variance of 47.4%, with a ‘women’s perception’ factor and a ‘social environment’ factor (Table 2). The component correlations between the two factors were found to be smaller than 0.30 (0.04) with direct oblimin rotation, therefore varimax rotation was used. Items 4 and 6 loaded on both factors with a difference smaller than 0.20 and were therefore eliminated. Reliability analyses showed a Cronbach’s alpha of 0.79 for the six-item women’s perception subscale, which increased to 0.84 after deletion of items 1 and 3. The three-item social environment subscale had a Cronbach’s alpha of 0.60, and the total seven-item LPRAQ-p had a Cronbach’s alpha of 0.77.

**Table 1:** Characteristics of two samples of third trimester pregnant women (*N* = 861)

	Sample I (*N* = 429)				Sample II (*N* = 432)			
*Demographics*	N	%	Mean (SD)	Range	N	%	Mean (SD)	Range
Age			30.2 (3.5)	20–40			30.2 (3.7)	19–43
High level of education	273	65.6			272	65.1		
Paid job	377	90.2			393	93.3		
Living with partner	413	98.6			418	99.1		
*Pregnancy related*								
Multiparity	199	46.9			205	48.5		
Unplanned pregnancy	23	5.5			27	6.4		
Problems with previous delivery	71	17.0			81	19.2		
*Lifestyle features*								
Smoking at 32 weeks	15	3.5			15	3.5		
Alcohol intake at 32 weeks	14	3.3			13	3.0		
*Psychological features*								
Depression earlier in life	67	16.1			59	14.0		
EDS at 32 weeks			5.0 (4.1)	0–20			5.0 (4.2)	0–22
TPDS-NA at 32 weeks			6.7 (4.6)	0–26			6.6 (4.6)	0–30
TPDS-NA worries about delivery at 32 weeks			2.8 (2.7)	0–14			2.5 (2.4)	0–13

Note: *SD* standard deviation; High level of education, Bachelor’s or Master’s degree; *EDS* Edinburgh Depression Scale; *TPDS-NA* Negative Affect subscale of the Tilburg Pregnancy Distress Scale

**Table 2:** Two-factor solution from explorative factor analysis (EFA) with varimax rotation in 429 (sample I) women who completed the 13-item LPRAQ-p

	Factor I *women’s perception*	Factor II *social environment*
Eigenvalue	3.4	1.8
Percentage of variance explained	30.7	16.7
**1. Pain relief makes me feel like I am not fully in control during labor**	.**66**	
2. Pain is part of the labor process		
**3. Pain relief during labor means that medication is administered, which can affect my baby**	.**45**	
4. The fact that receiving medication for pain relief means that I have to give birth in the hospital, keeps me from asking for it	0.45	− 0.33
5. I think too little attention is paid to the possible pros and cons of pain relief		
6. Pain during labor will strengthen the bond with my baby	0.50	− 0.37
**7. Because my pregnancy has already had a big impact on my body, I think it is normal to ask for pain relief**	.**70**	0.27
**8. I also ask for pain relief because of my partner**		.**72**
**9. I am convinced that if I get pain relief, I will feel much more self-confident during labor**	.**72**	0.44
**10. Pain relief will help me perform much better during labor**	.**72**	0.34
**11. My partner plays an important role in the decision to ask for pain relief during labor**		.**65**
**12. Pain during labor is outdated**	.**67**	0.33
**13. My (social) environment (friends, relatives) plays an important role in the decision to ask for pain relief during labor**		.**65**

Note: Items 2 and 5 were eliminated based on face validity. To retain items (bold**, ***n* = 9) a cut-off score of item loading of 0.40 was used and a minimum difference of 0.20 if an item had two loadings. Total explained variance is 47.4%

Subsequently, CFA was performed on the seven-item LPRAQ-p in the second sample, and showed a moderate model fit (CFI = 0.97, NFI = 0.95, TLI = 0.95, RMSEA = 0.08, lower bound = 0.05). However, item 7 showed poor standardized residual co-variances. After removing this item, a two-factor structure with six items showed an excellent model fit (CFI = 0.99, NFI = 0.98, TLI = 0.99, RMSEA = 0.02, lower bound = 0.01). EFA with varimax rotation was repeated in sample II on the six-item LPRAQ-p, again resulting in a two-factor structure explaining 65.9% of the variance (Table 3). The Cronbach’s alpha was 0.78 for the three-item women’s perception subscale, 0.67 for the three-item social environment subscale, and 0.75 for the total LPRAQ-p. The items were recoded from 1 -5 to 0–4, with total scores ranging from 0 to 24. Higher scores indicated greater willingness for request of pain relief medication during labor. Table 4 shows the LPRAQ-p.

**Table 3:** Final six-item LPRAQ-p with two-factor solution from factor analysis with varimax rotation in 432 (sample II) women with excellent model fit in confirmatory factor analysis (CFA)

	Factor I *women’s perception*	Factor II *social environment*
Eigenvalue	2.7	1.3
Percentage of variance explained	45.0	21.0
8. I also ask for pain relief because of my partner		0.78
9. I am convinced that if I get pain relief, I will feel much more self-confident during labor	0.88	
10. Pain relief will help me perform much better during labor	0.87	
11. My partner plays an important role in the decision to ask for pain relief during labor		0.77
12. Pain during labor is outdated	0.71	
13. My (social) environment (friends, relatives) plays an important role in the decision to ask for pain relief during labor		0.75

Note: CFI = 0.99; NFI = 0.98; TLI = 0.99; RMSEA = 0.02; lower bound = 0.01

**Table 4:** The Labor Pain Relief Attitude Questionnaire for pregnant women (LPRAQ-p)

	Completely disagree (1)	Disagree (2)	Neutral (3)	Agree (4)	Fully agree (5)
1. I also ask for pain relief because of my partner	□	□	□	□	□
2. I am convinced that if I get pain relief, I will feel much more self-confident during labor	□	□	□	□	□
3. Pain relief will help me perform much better during labor	□	□	□	□	□
4. My partner plays an important role in the decision to ask for pain relief during labor	□	□	□	□	□
5. Pain during labor is outdated	□	□	□	□	□
6. My (social) environment (friends, relatives) plays an important role in the decision to ask for pain relief during labor	□	□	□	□	□

Note: Subscale women’s perception = item 2, 3, 5. Subscale social environment = item 1, 4, 6. All items recoded from 1–5 to 0–4

### Concurrent and construct validity analyses

For concurrent and construct validity analyses, both samples were merged (*N* = 861). Skewness and kurtosis values showed a normal distribution of the six-item scale scores. As shown in Table 5, total EDS scores were significantly associated with the total LPRAQ-p scores (*r* = 0.133, *p* < 0.001) as well as with the scores on the women’s perception subscale (*r* = 0.134, *p* < 0.001) and social environment subscale (*r* = 0.076, *p* = 0.025), all small effect sizes. The TPDS-NA subscale scores and the worries about delivery subcomponent scores were significantly associated with the total LPRAQ-p scores with small to medium effect sizes (*r* = 0.223 to 0.250, all *p* < 0.001). The TPDS-NA and worries about delivery scores were also significantly associated with the women’s perception subscale scores with small to medium effect sizes (*r* = 0.243 to 0.267, all *p* < 0.001), and social environment subscale scores with small effect sizes (*r* = 0.104 to 0.125, *p* = 0.002 and *p* < 0.001, respectively).

**Table 5:** Correlation matrix with the six-item Labor Pain Relief Attitude Questionnaire for pregnant women (LPRAQ-p) and the third trimester symptoms of depression, pregnancy-specific distress and worries about delivery (*N* = 861)

	1.	2.	3.	4.	5.	6.
1. LPRAQ-p	1	0.86***	0.76***	0.13***	0.22***	0.25***
2. LPRAQ-p: women’s perception	-	1	0.33***	0.13***	0.24***	0.26***
3. LPRAQ-p: social environment	-	-	1	0.08*	0.10**	0.13***
4. EDS	-	-	-	1	0.51***	0.41***
5. TPDS-NA	-	-	-	-	1	0.87***
6. TPDS-NA worries about delivery	-	-	-	-	-	1

Note: *LPRAQ-p* Labor Pain Relief Attitude Questionnaire for pregnant women, higher scores indicate greater willingness for request of pain relief medication during labor; *EDS* Edinburgh Depression Scale; *TPDS-NA* Negative Affect subscale of the Tilburg Pregnancy Distress Scale

*p < 0.05, **p < 0.01, ***p < 0.001 (two-tailed)

**Fig. 1:**
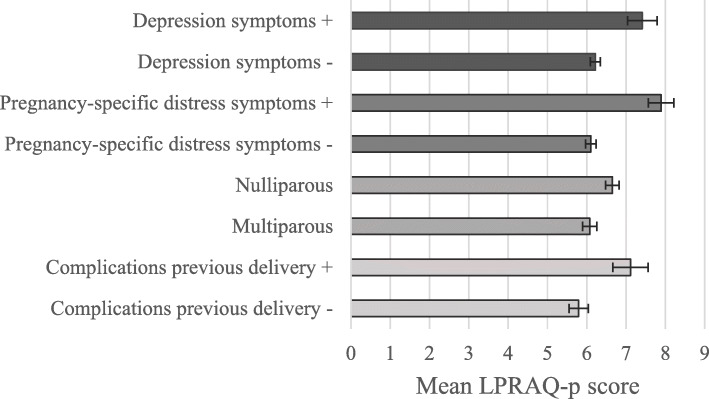
Differences in mean total scores of the Labor Pain Relief Attitude Questionnaire for pregnant women *(LPRAQ-p, higher scores indicate greater willingness for request of pain relief medication during labor)* between women with and without elevated levels of depression symptoms (***p*** **= .003**), between women with and without elevated levels of pregnancy-specific distress symptoms (***p*** **< 0.001**), between nulliparous and multiparous women (***p*** **= 0.022**), and between multiparous women with and without a history of complications during a previous delivery (***p*** **= 0.011**)

Next, construct validity was assessed by hypothesis testing. A graphical overview of the results regarding our hypotheses is shown in Fig. 1. Our first hypothesis was that women with elevated levels of depression and pregnancy-specific distress symptoms scored higher on the LPRAQ-p than women without elevated levels of depression and pregnancy-specific distress symptoms. In the third trimester of pregnancy, 125 (14.5%) of the 861 women scored above the EDS cut-off score (EDS ≥ 10), a commonly used cut-off in this trimester to define elevated levels of depression symptoms. In the current study, this cut-off referred to the 86th percentile of EDS scores. Women with elevated levels of depression symptoms had significantly greater willingness for request of pain relief medication during labor, compared to women without elevated levels of depression symptoms (t(156) = 3.00, *p* = 0.003, small effect size). Regarding pregnancy-specific distress symptoms, the cut-off score for the TPDS-NA was defined using a similar cut-off as for the EDS: the 86th percentile, which resulted in a cut-off score of 11 for the TPDS-NA. In total, 141 (16.4%) women scored above the TPDS-NA cut-off score, categorized as elevated levels of pregnancy-specific distress symptoms. These women scored significantly higher on the LPRAQ-p, compared to women without elevated levels of pregnancy-specific distress symptoms (t(859) = 5.37, *p* < 0.001, medium effect size).

Our second hypothesis was that nulliparous women had greater willingness for request of pain relief medication during labor than multiparous women. Indeed, nulliparous women scored significantly higher on the LPRAQ-p compared to multiparous women (t(845) = 2.29, *p* = 0.022, small effect size). Our last hypothesis was that multiparous women with a history of complications during a previous delivery scored higher on the LPRAQ-p than multiparous women without a history of complications during a previous delivery. Of the sample of pregnant women, 402 were multiparous. These women were asked for possible complications during a previous delivery. A total of 245 women (62.5%) reported no complications: group (1). Sixty-nine women (17.6%) reported complications regarding poor progression of labor (such as delayed dilation phase, secondary Caesarean section, use of ventouse or forceps and fetal hypoxia): group (2). Seventy-eight women (19.9%) reported miscellaneous complications (such as prolonged second stage of labor, primary Caesarean section and fetus in breech position): group (3). When comparing LPRAQ-p scores between group 1 and 2, women who reported no complications had a significantly lower score (t(312) = 2.57, *p* = 0.011, small effect size).

We finally performed a linear regression analysis with LPRAQ-p scores as dependent variable and depression symptoms and pregnancy-specific distress symptoms as independent variables, adjusted for covariates (age, level of education and parity). The total model, including five predictors, was significant (F(5, 823) = 12.18, *p* < 0.001) and explained 6.9% of the variance in willingness for request of pain relief medication during labor. The most important association was found for pregnancy-specific distress symptoms, with the highest standardized beta of 0.20 (*p* < 0.001), as shown in Table 6. Also, higher age (β = 0.11, *p* = 0.002) and nulliparity (β = − 0.09, *p* = 0.015) were significantly related to higher scores on the LPRAQ-p. A high level of education was related to lower willingness for request of pain relief medication during labor (β = − 0.11, *p* = 0.003).

**Table 6:** Multiple regression predicting labor pain relief attitude (N = 861)

	B (SE)	β	*T*	95% CI
**Step 1**				
Age	0.10 (0.04)	− 0.11	2.75**	[0.03, 0.18]
High level of education	− 0.87 (0.28)	0.10	-3.11**	[-1.41, − 0.32]
Multiparity	− 0.82 (0.26)	− 0.11	-3.13**	[-1.34, − 0.31]
Depression symptoms	0.12 (0.03)	0.13	3.84***	[0.06, 0.18]
**Step 2**				
Age	0.11 (0.04)	0.11	3.06**	[0.04, 0.19]
High level of education	− 0.82 (0.27)	− 0.11	-2.98**	[-1.36, − 0.28]
Multiparity	− 0.64 (0.26)	− 0.09	-2.44*	[-1.15, − 0.12]
Depression symptoms	0.03 (0.04)	0.03	0.78	[-0.04, 0.10]
Pregnancy-specific distress symptoms	0.16 (0.03)	0.20	5.06***	[0.10, 0.22]

Note: labor pain relief attitude, measured with the Labor Pain Relief Attitude Questionnaire for pregnant women (LPRAQ-p), higher scores indicate greater willingness for request of pain relief medication during labor; *B* unstandardized regression coefficient; *SE* standard error; *β* standardized regression coefficient; *CI* Confidence Interval; High level of education, Bachelor’s or Master’s degree

*p < 0.05, **p < 0.01, *** *p* < 0.001

## Discussion

The current study aimed to develop and validate a questionnaire that measures a pregnant woman’s attitude towards labor pain relief. The LPRAQ-p was developed based on the outcome of focus group interviews. Subsequent validation analyses using EFA and CFA showed that the six-item LPRAQ-p has good psychometric properties: a two-factor structure with acceptable to good internal consistency and excellent model fit.

A Cronbach’s alpha between 0.60 and 0.70 indicates an acceptable level of reliability and a Cronbach’s alpha between 0.70 and 0.80 a good level [35]. Both the women’s environment subscale and the total LPRAQ-p have good reliability scores (all above 0.75), while the social environment subscale has acceptable reliability scores (above 0.60). It must be noted that a recommended Cronbach’s alpha of ≥ 0.70 has been described as well [24]. However, Cronbach’s alpha is very sensitive to the number of items and especially in an ultra-short scale this cut-off of 0.70 can be regarded as arbitrary [36].

Results showed that the LPRAQ-p consists of two subscales, including a women’s perception subscale. Two items of this subscale refer to beliefs a woman can have towards the outcome of receiving EA: the belief that she feels more self-confident during labor (item 9) and performs much better (item 10) with EA. Remarkably, the confidence women have in their ability to cope with labor is of major importance to how they perceive pain during labor [37, 38]. This self-efficacy for labor has been related to decreased pain perception during labor [39, 40]. This could imply that a woman’s belief of not being able to cope with labor without EA involves an enhanced pain perception during labor, which increases the willingness for request of EA even further. Indeed, confidence in the ability to cope with labor has been related to decreased pain medication use during labor [41]. The third item of the women’s perception subscale comprises a general belief towards pain in labor (item 12), and addresses how women anticipate labor pain. Women can approach labor as a medical event with risks or as a normal and natural process [42]. When viewing labor as a medical event, it is more likely that women wish to eradicate the pain with EA. However, when women view labor as a natural process they are more willing to embrace the pain, which enhances the coping ability [43–45]. An interesting paradox in these women is that despite its challenging nature, they need the pain because it facilitates birth and therefore the joy and happiness of meeting their baby [46].

The second subscale of the LPRAQ-p is the social environment subscale, containing two partner items and one item concerning family and friends. Item 8 involves the influence of the anticipated partner’s needs during labor. For a partner, the major challenge during labor is to see his loved one suffering from pain [47], which can make the partner feel frustrated and helpless [48]. However, being helpful is what a woman in labor needs most from her partner [49]. Receiving EA has been associated with decreased partner anxiety and stress and an increased partner support and helpfulness during labor [50]. This implies that a woman may have greater willingness for request of pain relief medication during labor for both her partner’s wellbeing and her own need of support. The other two items of the social environment subscale refer to influences of the attitudes towards labor pain relief of respectively the partner (item 11), and family and friends (item 13). Interestingly, a previous study found that a partner’s preference for EA was related to receiving EA, while a partner’s preference for labor without EA had no effect [16]. In addition, being encouraged by family and friends to ask for EA was reported as a reason that pregnant women had greater willingness for request of pain relief medication during labor [16]. Moreover, family members and friends with children can influence childbirth expectations by providing stories about their own childbirth experiences [42, 51]. Especially the stories regarding bad experiences may enhance a woman’s willingness for request of pain relief medication during labor [42].

With regard to concurrent validity, the LPRAQ-p and its subscales were significantly correlated with symptoms of depression, pregnancy-specific distress and worries about delivery. Moreover, regarding construct validity all our hypotheses were confirmed. Women with elevated levels of depression and pregnancy-specific distress symptoms showed significantly higher scores on the LPRAQ-p. Our multiple linear regression analysis showed that pregnancy-specific distress symptoms predicted greater willingness for request of pain relief medication during labor, controlled for depression symptoms, age, level of education and parity. These findings correspond to previous studies reporting an association between antenatal fear of childbirth and request for labor pain relief [12–14]. Fear of childbirth has been related to fear of pain [52] and lower childbirth self-efficacy [53], and both seem to be important factors in a woman’s attitude towards labor pain relief. This also applies to negative experiences provided by others, which has been related to fear of childbirth as well [54]. Furthermore, a link between fear of childbirth and negative mood has been reported [54], which may explain our results regarding depression symptoms. Moreover, depression has been related to expectations for negative outcomes in general [18, 19]. It could be speculated that women with depression symptoms are more likely to approach labor as a medical event with risks, with greater willingness to suppress the pain. However, our findings with regard to multiple linear regression showed that depression symptoms were no longer associated with greater willingness for request of pain relief medication during labor when pregnancy-specific distress symptoms were entered in the model. This could be explained by the high correlation between depression symptoms and pregnancy-specific distress symptoms (*r* = 0.51, *p* < 0.001, large effect size, see Table 5), suggesting that pregnancy-specific distress symptoms are more important in predicting a woman’s willingness for request of pain relief medication during labor. It should be noted that the TPDS-NA measures worry symptoms about pregnancy and delivery, with examples of items concerning worries about delivery being: “I worry about the delivery”, “I am afraid I will lose self-control during delivery” and “The delivery is troubling me”. It seems reasonable to believe that scores on the TPDS-NA are more predictive of a pregnant woman’s willingness for request of pain relief medication during labor than scores on a general depression questionnaire such as the EDS.

Furthermore, with regard to construct validity, we found at both a univariate and multivariate level, that nulliparous women had greater willingness for request of pain relief medication during labor, which was also reported in previous studies [10, 17]. It is likely that multiparous women have more confidence in their ability to cope with labor, because they succeeded in giving birth before. Indeed, a recent study found that multiparous women reported higher childbirth self-efficacy scores compared to nulliparous women [53]. Multiparous women with a history of complications during a previous delivery had greater willingness for request of pain relief medication during labor. These women can have expectations that something similar will happen during the next delivery [42]. This may contribute to an enhanced childbirth fear [55] and likelihood of approaching labor as a medical event [42], and could therefore increase the preference for a pain free delivery.

Finally, our linear regression model predicted only a small percentage of the variance in scores on the LPRAQ-p, which should be taken into account when interpreting the results. Besides pregnancy-specific distress symptoms and nulliparity, a higher age was significantly associated with greater willingness for request of pain relief medication during labor and a high education level with lower willingness for request of pain relief medication during labor. Previous studies found different results regarding the association of age and education level with the request for EA during labor. Some studies found no significant association for both age and education level [56, 57]. Other studies reported a similar relation between age and receiving EA [16, 17], but an opposite relation between education level and receiving EA [16, 58, 59].

A major strength of the current study is that the development of the LPRAQ-p was based on direct input from pregnant women, new mothers and obstetric caregivers using focus group interviews. Other strengths include the large sample size and the performance of both EFA and CFA in different samples to validate the questionnaire. A limitation of the current study is that the participants were white Dutch women, while in the Netherlands up to 25% of the women have a migration background (11% Western and 14% non-Western) [60]. Since cultural background influences a woman’s attitude towards labor pain relief [4, 9], it is important to re-evaluate the psychometric properties of the LPRAQ-p in women of other ethnic groups. Also, the number of highly educated women was slightly higher in the current study compared to the national figures (65% versus 55%) [61], which may limit the generalizability of the results. With regard to the *t*-tests, mostly small effect sizes were found, which suggests that possible differences should be interpreted with caution.

The concept of the developed LPRAQ-p seems to be clinically relevant. It is important to have knowledge of a pregnant woman’s attitude towards labor pain relief, since EA can have detrimental effects on a woman in labor. Receiving EA has been related to an extended first and second stage of labor, increased need for additional oxytocin, hypotension, motor blockade, fever and urinary retention [20], and length of exposure to EA has been associated with non-spontaneous deliveries [21]. During the antenatal period, women often already plan to ask for EA during labor [9, 10]. Up until this point, there was no validated measure to obtain a pregnant woman’s attitude towards labor pain relief. The LPRAQ-p could be a valuable screening instrument to identify pregnant women with greater willingness for request of pain relief medication during labor. Because high scores can reflect poor self-efficacy for labor, and pregnancy-specific distress symptoms were significantly and independently related to high scores, these women may benefit from extra help and support during pregnancy and labor. During the focus group interviews, it was identified that self-efficacy for labor seems to be an important part of attitude towards labor pain relief as reflected in several final items of the LPRAQ-p. Moreover, our results suggest that pregnant women with elevated levels of pregnancy-specific distress symptoms have greater willingness for request of pain relief medication during labor. This means that strategies to help women with higher scores on the LPRAQ-p should address both self-efficacy and pregnancy-specific distress, especially since fear of childbirth has been associated with lower childbirth self-efficacy [53]. Strategies could particularly be useful for nulliparous women and multiparous women with a history of complications during a previous delivery, who both showed to have higher scores on the LPRAQ-p, and have previously been described to have lower childbirth self-efficacy [53] and enhanced fear of childbirth [55].

*During pregnancy*, an obstetric caregiver could advise women with higher scores on the LPRAQ-p to participate in a childbirth education course in order to strengthen their self-efficacy and to reduce pregnancy-specific distress symptoms. Childbirth self-efficacy has indeed been related to knowledge of labor and practical coping skills [62]. Several studies have examined the effectiveness of a childbirth education program during pregnancy on childbirth self-efficacy and fear of childbirth. A recent study examined the effectiveness of a companion-integrated childbirth preparation, designed to educate and support pregnant women and their birth companions, and found promising effects on fear of childbirth and childbirth self-efficacy [63]. Two randomized controlled trials on a childbirth psychoeducational program showed a reduction in fear of childbirth in the intervention group [64, 65]. One of these trials addressed self-efficacy as well, and reported a significant improvement [65]. A randomized controlled trial on a short mindfulness-based childbirth preparation course for pregnant women and their partners showed an improvement in childbirth-efficacy in the intervention group [66]. Moreover, a pilot study on a Mindfulness-Based Childbirth Education (MBCE) program found improvements in childbirth self-efficacy and fear of childbirth [67]. Future research should investigate which childbirth education programs are most suitable for women with greater willingness for request of pain relief medication during labor, and whether these programs are effective in reducing EA rates.

Knowing which pregnant women have higher scores on the LPRAQ-p could also help obstetric caregivers to decide upon offering continuous support *during labor*. Lack of partner or social support during pregnancy has been associated with lower childbirth self-efficacy [53] and fear of childbirth [68]. Therefore, it seems likely that support by a companion during labor has a beneficial effect on women with greater willingness for request of pain relief medication during labor. Continuous support during labor involves the constant presence of a companion during labor and delivery, who provides emotional and informational support, advices about coping techniques and comfort measures, and advocates on behalf of the woman in labor [69]. The companion could be a doula, the partner, a family member or a friend [69]. Continuous support is most helpful when it is provided by someone who is calm and trusted, with an accepting attitude and the ability to give a positive meaning to the pain [70]. According to a recent Cochrane review, women who had continuous support during labor were less likely to receive EA [69].

Interestingly, while one may expect that receiving pain relief medication during labor could enhance the childbirth experience, a prospective study found that women who wanted to avoid labor pain relief medication were more satisfied after the birth than women who received labor pain relief medication [71]. This study related fear of labor pain to a lower satisfaction with the childbirth experience [71]. Moreover, two systematic reviews reported that receiving labor pain relief medication had no effect on the childbirth experience [72, 73]. Instead, birth preparedness and continuous support were described to be important strategies to improve the experience of childbirth [72, 73]. This could imply that antenatal childbirth education and continuous support during labor could both reduce EA rates and enhance the childbirth experience.

## Conclusions

The current study showed that the six-item LPRAQ-p is a short, valid and user-friendly instrument with good psychometric properties. High scores on this questionnaire reflect greater willingness for request of pain relief medication during labor, and were highly correlated with pregnancy-specific distress symptoms. Childbirth self-efficacy seems to be an important part of attitude towards labor pain relief. Therefore, the LPRAQ-p may be a valuable screening instrument during pregnancy to detect women with lower childbirth self-efficacy and more pregnancy-specific distress symptoms, who potentially might benefit from additional support.

## Data Availability

The datasets generated and analyzed during the current study are available from the corresponding author on reasonable request.

## Abbreviations

CFA: Confirmatory factor analysis
CFI: Comparative fit index
EA: Epidural analgesia
EDS: Edinburgh depression scale
EFA: Exploratory factor analysis
HAPPY: Holistic approach to pregnancy and the first postpartum year
LPRAQ-p: Labor pain relief attitude questionnaire for pregnant women
MBCE: Mindfulness-based childbirth education
NFI: Normed fit index
RMSEA: Root mean square error of approximation
SD: Standard deviation
SPSS: Statistical package for social Sciences
TLI: Tucker-lewis index
TPDS: Tilburg pregnancy distress scale
TPDS-NA: Negative affect subscale of the tilburg pregnancy distress scale
